# Supplementary figures and images for: The clinicopathological significance of Thrombospondin-4 expression in the tumor microenvironment of gastric cancer
Source: PLoS One. 2019 Nov 8;14(11):e0224727. doi: 10.1371/journal.pone.0224727 (PMC6839882; doi:10.1371/journal.pone.0224727)

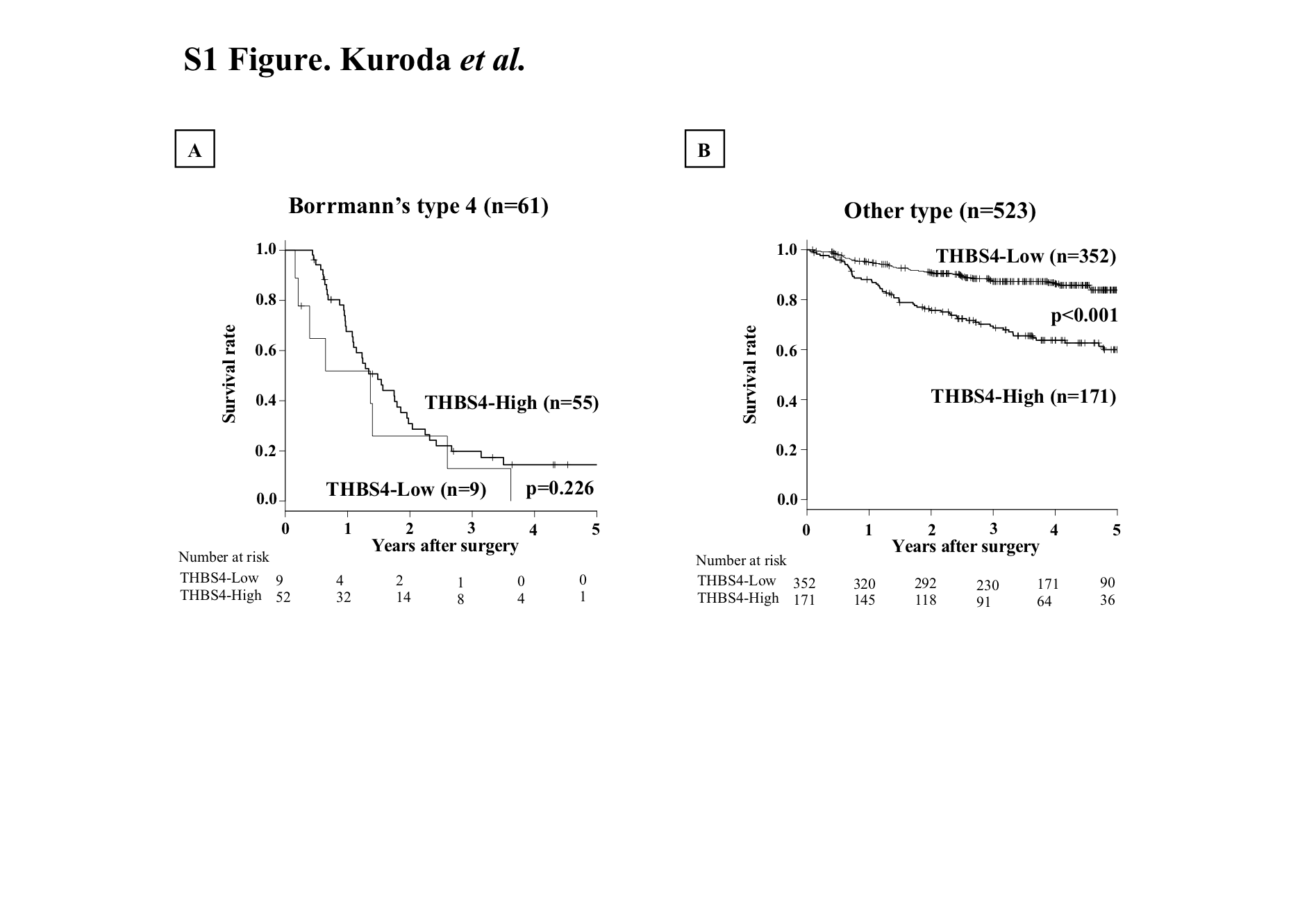

Supplement: S1 Fig — No significant difference in overall survival was shown between the THBS-high group and THBS-low group at Borrmann’s type 4. Whereas the prognosis of the patients with high THBS4 expression were poorer than that of the patients with low THBS4 expression at other type. (TIFF) [file pone.0224727.s001.tiff]

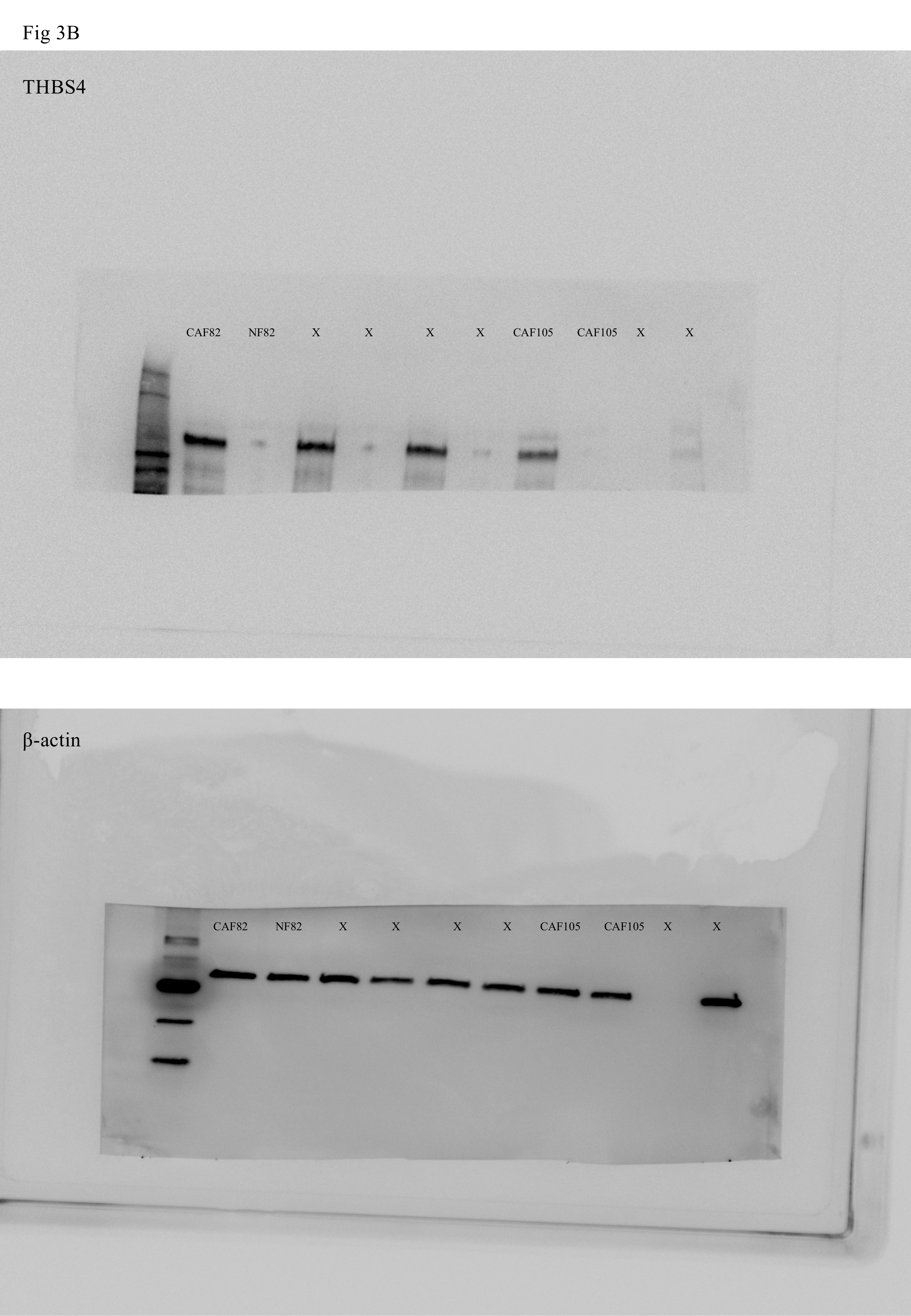

Supplement: S1 Raw Images — (TIFF) [file pone.0224727.s003.tiff]

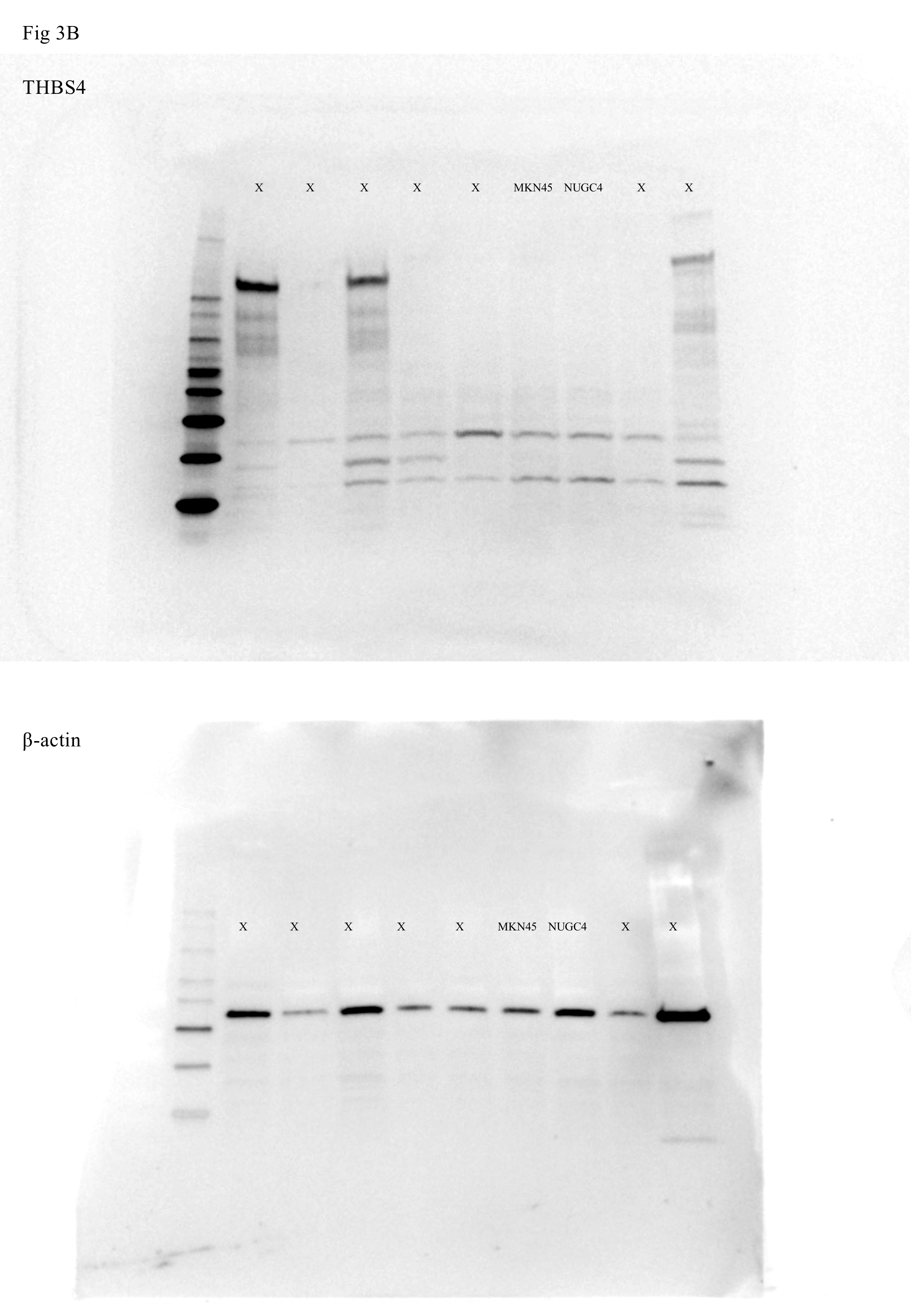

Supplement: S2 Raw Images — (TIFF) [file pone.0224727.s004.tiff]
