# Supplementary material for: The clinicopathological significance of Thrombospondin-4 expression in the tumor microenvironment of gastric cancer
Source: PLoS One. 2019 Nov 8;14(11):e0224727. doi: 10.1371/journal.pone.0224727 (PMC6839882; doi:10.1371/journal.pone.0224727)
Supplement: S1 Table — (DOCX) [file pone.0224727.s005.docx]

**S1 Table. Correlation between the expression of THBS4 in tumor stromal cells and clinicopathologic features in 61 type 4 tumors.**

|  | THBS4 | |  |
| --- | --- | --- | --- |
|  | High  (n=52, 85.2%) | Low  (n=9, 14.8%) | p value |
| Age (year-old) |  |  |  |
| < 65 | 25 (48.1%) | 4 (44.4%) |  |
| ≥ 65 | 27 (51.9%) | 5 (55.6%) | 1.000 |
| Gender |  |  |  |
| Female | 21 (40.4%) | 1 (11.1%) |  |
| Male | 31 (59.6%) | 8 (89.9%) | 0.138 |
| Tumor diameter |  |  |  |
| < 50 | 1 (1.9%) | 0 (0.0%) |  |
| ≥ 50 | 51 (98.1%) | 9 (100.0%) | 1.000 |
| Microscopic type |  |  |  |
| Differentiated | 4 (7.7%) | 2 (22.2%) |  |
| Undifferentiated | 48 (92.3%) | 7 (77.8%) | 0.212 |
| Depth of tumor invasion |  |  |  |
| T1-2 | 1 (1.9%) | 0 (0.0%) |  |
| T3-4 | 51 (98.1%) | 9 (100.0%) | 1.000 |
| Lymph node metastasis |  |  |  |
| N0 | 2 (3.8%) | 0 (0.0%) |  |
| N1-3 | 50 (96.2%) | 9 (100.0%) | 1.000 |
| Lymphatic invasion |  |  |  |
| Absent | 4 (7.7%) | 0 (0.0%) |  |
| Present | 48 (92.3%) | 9 (100.0%) | 1.000 |
| Venous invasion |  |  |  |
| Absent | 36 (69.2%) | 5 (55.6%) |  |
| Present | 16 (30.8%) | 4 (44.4%) | 0.458 |
| Ascites cytology |  |  |  |
| Negative | 26 (50.0%) | 5 (55.6%) |  |
| Positive | 26 (50.0%) | 4 (44.4%) | 1.000 |
| Peritoneal metastasis |  |  |  |
| Absent | 39 (75.0%) | 4 (44.4%) |  |
| Present | 13 (25.0%) | 5 (55.6%) | 0.108 |
| Hepatic metastasis |  |  |  |
| Negative | 50 (96.2%) | 8 (88.9%) |  |
| Positive | 2 (3.8%) | 1 (11.1%) | 0.386 |
| pStage |  |  |  |
| I, II | 1 (1.9%) | 0 (0.0%) |  |
| III, IV | 51 (98.1%) | 9 (100.0%) | 1.000 |
