# Supplementary material for: The clinicopathological significance of Thrombospondin-4 expression in the tumor microenvironment of gastric cancer
Source: PLoS One. 2019 Nov 8;14(11):e0224727. doi: 10.1371/journal.pone.0224727 (PMC6839882; doi:10.1371/journal.pone.0224727)
Supplement: S2 Table — (DOCX) [file pone.0224727.s006.docx]

**S2 Table. Correlation between the expression of THBS4 in stromal cells and clinicopathologic features in type 0-3 tumors.**

|  | THBS4 | |  |
| --- | --- | --- | --- |
|  | High  (n=171, 32.7%) | Low  (n=352, 67.3%) | p value |
| Age (year-old) |  |  |  |
| < 65 | 80 (46.8%) | 147 (41.8%) |  |
| ≥ 65 | 91 (53.2%) | 205 (58.2%) | 0.301 |
| Gender |  |  |  |
| Female | 76 (44.4%) | 161 (45.7%) |  |
| Male | 95 (55.6%) | 191 (54.3%) | 0.852 |
| Tumor diameter |  |  |  |
| < 50 | 103 (60.2%) | 250 (71.0%) |  |
| ≥ 50 | 68 (39.8%) | 102 (29.0%) | <0.001 |
| Microscopic type |  |  |  |
| Differentiated | 77 (45.0%) | 203 (57.7%) |  |
| Undifferentiated | 94 (55.0%) | 149 (42.3%) | 0.017 |
| Depth of tumor invasion |  |  |  |
| T1-2 | 72 (42.1%) | 264 (75.0%) |  |
| T3-4 | 99 (57.9%) | 88 (25.0%) | <0.001 |
| Lymph node metastasis |  |  |  |
| N0 | 81 (47.4%) | 246 (70.1%) |  |
| N1-3 | 90 (52.6%) | 105 (29.9%) | <0.001 |
| Lymphatic invasion |  |  |  |
| Absent | 52 (30.4%) | 204 (58.0%) |  |
| Present | 119 (69.6%) | 148 (42.9%) | <0.001 |
| Venous invasion |  |  |  |
| Absent | 140 (81.9%) | 305 (85.2%) |  |
| Present | 31 (18.1%) | 56 (14.8%) | 0.372 |
| Ascites cytology |  |  |  |
| Negative | 148 (86.5%) | 341 (96.9%) |  |
| Positive | 23 (13.5%) | 11 (3.1%) | <0.001 |
| Peritoneal metastasis |  |  |  |
| Absent | 161 (94.2%) | 345 (98.0%) |  |
| Present | 10 (5.8%) | 7 (2.0%) | 0.032 |
| Hepatic metastasis |  |  |  |
| Negative | 166 (97.1%) | 346 (98.1%) |  |
| Positive | 5 (2.9%) | 6 (1.9%) | 0.351 |
| pStage |  |  |  |
| I, II | 98 (57.3%) | 285 (81.0%) |  |
| III, IV | 73 (42.7%) | 67 (19.0%) | <0.001 |
