# Supplementary material for: The clinicopathological significance of Thrombospondin-4 expression in the tumor microenvironment of gastric cancer
Source: PLoS One. 2019 Nov 8;14(11):e0224727. doi: 10.1371/journal.pone.0224727 (PMC6839882; doi:10.1371/journal.pone.0224727)
Supplement: S3 Table — (DOCX) [file pone.0224727.s007.docx]

**S3 Table.** **Recurrence pattern according to the stage.**

| Recurrence type | Stage II | Stage III |
| --- | --- | --- |
| Hematogenous metastasis | 9 (69.2%) | 18 (36.7%) |
| Peritoneal metastasis | 3 (23.1%) | 19 (38.8%) |
| Lymph node metastasis | 1 (7.7%) | 10 (20.4%) |
| Local recurrence | 0 (0%) | 2 (4.1%) |
| Total | 13 (100%) | 49 (100%) |
