# Supplementary material for: The clinicopathological significance of Thrombospondin-4 expression in the tumor microenvironment of gastric cancer
Source: PLoS One. 2019 Nov 8;14(11):e0224727. doi: 10.1371/journal.pone.0224727 (PMC6839882; doi:10.1371/journal.pone.0224727)
Supplement: Attachment — Submitted filename: Response to Reviewers.docx [file pone.0224727.s008.docx]

September 19, 2019

Professor Aamir Ahmad
Academic Editor

*PLOS ONE*

Dear Professor Aamir Ahmad;

**Re; Manuscript:** PONE-D-19-19270

Title: The Clinicopathological Significance of Thrombospondin-4 Expression in the Tumor Microenvironment of Gastric Cancer

Authors: Kenji Kuroda, et al.

We greatly appreciate your invitation for us to revise our article “The Clinicopathological Significance of Thrombospondin-4 Expression in the Tumor Microenvironment of Gastric Cancer”. We would like to thank you for a number of comments and suggestions for improvement in our manuscript. We have carefully considered the referees’ comments and have made point-by-point responses as described below. Also, we highlight all changes in the revised manuscript. This manuscript is not being considered in whole or in part by any other journal. All authors are aware of the content of this manuscript.

The following is the funding information regarding to this manuscript;

This study is partially supported by Japan Society for the Promotion of Science KAKENHI (Grant-in-Aid for Scientific Research B; Grant Number JP18H02883 (M.Y.)). (<https://www.jsps.go.jp/english/e-grants/>)

The funder had no role in study design, data collection and analysis, decision to publish, or preparation of the manuscript. There was no additional external funding received for this study.

We hope you will seriously consider this report for publication in *PLOS ONE*.

Sincerely,

Masakazu Yashiro, MD

Molecular Oncology and Therapeutics, Osaka City University Graduate School of Medicine,

1-4-3 Asahi-machi, Abeno-ku, Osaka 545-8585, Japan

TEL; (+81) 6-6645-3838

FAX; (+81) 6-6646-6450

**Reviewer #1**

Thank you very much for the careful review of the Reviewer #1. We corrected several points according to the descriptions by the Reviewer #1, as described below. We indicated the changes point by point and highlighted them in the revised paper.

1. Authors determined the expression of THBS4 in gastric cancer tissues according to the intensity and the percentage of stained cancer cells and stromal cells (Page 4, Line 71). However, they said that it was only expression in stromal cells. How did authors calculate the percentage of positive THBS cells? Was the percentage meaning the ratio of positive stromal cells in all cells including cancer cells and stromal cells?

We calculated the percentage of positive THBS cells by the ratio of stained stromal cells in all stromal cells. We added the comments in the materials and methods. **(on page4 line 22-26)**

2. In figure 1, the positive staining of THBS4 look like too diffuse in the entire tissues. Can authors show the clearer photo for THBS4 staining?

We replaced the picture with the clearer photo for THBS4 staining in Figure 1.

3. The gastric cancer generally showed histologic heterogeneity according to the classification. In figure 1, I recommend that authors show the representative photos for THBS4 expression in two different histological subtypes (diffuse and intestinal type).

We added the picture of THBS4 expression in two different histological subtypes including diffuse type and intestinal type. We added some comments in the manuscript. **(on page 7 line 12-13)**

4. In figure 1, the staining photo of cytokeratin, cancer cell specific maker, should be added to support that THBS4 was not stained in cancer cells.

We evaluated the expression of cytokeratin as cancer cell specific maker in the gastric cancer tissues. Cancer cells were positive for cytokeratin staining, but negative for THBS4 staining. The pictures of cytokeratin were added in Figure 1. We added these comments in the manuscript. **(on page 2 line 15)** **(on page 4 line 17) (on page 7 line 5-6) (on page 7 line 12) (on page 7 line 15)**

5. In figure 3, authors just showed the expression of THBS4 in NAF and CAF. I recommend authors that the results should be added the expression of THBS4 of several gastric cancer cell lines.

In accordance with the Reviewer’s comment, we added western blot of THBS4 of two gastric cancer cell lines, MKN45 and NUGC4 in Figure 3B. We added some comments in the manuscript. **(on page 2 line 13) (on page 2 line 17) (on page 5 line 9-10**) **(on page 11 line 12**) **(on page 11 line 18**) **(on page 12 line 3-7**)

6. Authors described in abstract and conclusion that “THBS4 from CAFs is associated with the metastasis of cancer cells” and “THBS4 from CAFs might be associated with the invasion of cancer cells”. However, they did not show the any data for proof of concept. Authors should add the experimental data to prove them, or the descriptions in abstract and conclusion should be toned down.

We showed in the results that THBS4-high expression in stroma was significantly correlated with higher tumor invasion (p<0.001), lymph node metastasis (p<0.001), lymphatic invasion (p<0.001), peritoneal cytology (p<0.001), and peritoneal metastasis (p<0.001). Taken together, we found that the prognosis of patients with high THBS4 expression in tumor stroma was significantly poorer than that of patients with low THBS4 expression. These findings indicated that THBS4 from CAFs might be associated with the invasion and metastasis of cancer cells. We added these comments in the discussion. **(on page 12 line 12-20)**

7. Authors emphasized that THBS4 is especially expression on CAFs of macroscopic type-4 gastric cancer. It would be better to add the results of subgroup analysis for type-4 or other type gastric cancer.

We performed the subgroup analysis of type-4 or other types gastric cancer. No significant difference of clinic-pathological factors was found between THBS4-high group and THBS4-low group in Bormann’s type 4 cancer (S1 Fig and S1 Table). In contrast, the other types gastric cancer had significant differences between the two groups. (S1 Fig and S2 Table). THBS4 might be associated with Bormann’s type 4 cancer, but not associated with malignant progression of Bormann’s type 4 cancer. We added these comments in the manuscript. **(on page 7 line 25-28) (on page 9 line 6-7)**

8. Authors insisted that non-significance of stage II would be explained as recurrent pattern and adjuvant chemotherapy. To supper this hypothesis, recurrent pattern or adjuvant regimen according to the stage should be added.

As reviewer pointed out, we added S3 Table which shows recurrence site according to the stage. Hematogenous recurrence was frequent in patients at stage II patients, in compared with those at stage III. In contrast, it was difficult to compare adjuvant regimen according to the stage, because the standard regimen for adjuvant chemotherapy was not established for the enrolled patients at the time. We added these comments in the text.

**(on page 9 line 10; on page 9 line 13)**

**Reviewer #2**

Thank you very much for the careful review of the Reviewer #2. We correct several points according to the descriptions by the Reviewer #2, as follows.

1. The authors used α-SMA to indicate the location of CAFs. However, α-SMA is a microfilament protein with contractile ability, mainly expressed at myoepithelial cells. Although α-SMA expression could also appear in the transformation of fibroblasts to myofibroblasts around cancer cells, the positive rate of α-SMA in CAFs is not high, and some of muscle tissues may be unexpectedly positive. I recommend the authors to add podoplanin (PDPN) in immunostaining assays, which is a widely accepted marker for CAFs with a relative high expression level.

I agree with the Reviewer’s comment, we added the immunostaining experiment of podoplanin which evaluated the for CAF in tumor. The picture of podoplanin staining was added in Fig 1. And we commented the Podoplanin immunostaining in the text. **(on page 2 line 15) (on page 4 line 11-20) (on page 7 line 8) (on page 7 line 15) (on page 12 line 5)**.

2. Primary culture was performed to obtain CAFs and NAFs in this research. However, there was no demonstrations or photos to show the status of cells in order to verify the results. And the isolations and purifications of CAFs and NAFs were not mentioned either.

We added representative pictures of CAFs and NFs by Podoplanin staining in Fig 3A. The method for the primary culture of CAFs and NFs was added in the manuscript. CAFs and NFs were defined by αSMA staining, as previously reported. **(on page 5 line 4-9)** **(on page 5 line 12-16)**

1. Fuyuhiro Y, Yashiro M, Noda S, Matsuoka J, Hasegawa T, Kato Y, et al. Cancer-associated orthotopic myofibroblasts stimulates the motility of gastric carcinoma cells. Cancer Sci. 2012;103(4):797-805.

2. Fuyuhiro Y, Yashiro M, Noda S, Kashiwagi S, Matsuoka J, Doi Y, et al. Myofibroblasts are associated with the progression of scirrhous gastric carcinoma. Exp Ther Med. 2010;1(4):547-51.

3. Inconsistent data were found between abstract and main text (e.g. positive rate of THBS4 expression).

We corrected them. **(on page 2 line 11)**

4. For WB assay, the authors did not elucidate the sample size and necessary statistical analysis (t-test). A solitary result cannot clarify the real world.

We added statistical analysis of western blot of THBS4 in CAFs and NFs in Fig 3B. We added the comments in the results. **(on page 11 line 9-12) (on page 11 line 17-18).**

5. According to the clinical data, THBS4 in stroma has obvious correlations with tumor invasion and lymph node metastasis. Since the primary CAFs have been cultured successfully, the authors should perform gene function experiments (e.g. migration & invasion) subsequently and explore the potential mechanisms to explain the phenomena they observed.

We agree that additional experiments on function of THBS4 would be valuable, as reviewer commented. We plan to investigate THBS4 function in CAFs, and submit as a new paper of *in vitro* experiment in the near future. Then, we added these comments in the discussion, as follows. Muppala S et al. reported that THBS4 is upregulated in response to TGFβ and promotes the effect of TGFβ on angiogenesis, mediating cell proliferation and survival [3]. Forester et al. showed *THBS4* transcription on CAFs was stimulated by incubation with conditioned medium derived from human diffuse gastric cancer lines [4]. It was speculated that TGF-β derived from cancer cells stimulated THBS4 expression on CAFs resulting in cancer proliferation and progression. We added these comments in the text. **(on page 12 line 23-29)**

3. Muppala S, Xiao R, Krukovets I, Verbovetsky D, Yendamuri R, Habib N, et al. Thrombospondin-4 mediates TGF-beta-induced angiogenesis. Oncogene. 2017;36(36):5189-98.

4. Forster S, Gretschel S, Jons T, Yashiro M, Kemmner W. THBS4, a novel stromal molecule of diffuse-type gastric adenocarcinomas, identified by transcriptome-wide expression profiling. Mod Pathol. 2011;24(10):1390-403.

6. Are there any references to support the IHC evaluating method of THBS4 expression? If yes, please cite the articles, otherwise the authors should state the reason why score 3 was the watershed.

We cited a reference which supports our IHC evaluating method of Thrombospondin4 (THBS4), as described below.

5. Huang T, Liu D, Wang Y, Li P, Sun L, Xiong H, et al. FGFR2 Promotes Gastric Cancer Progression by Inhibiting the Expression of Thrombospondin4 via PI3K-Akt-Mtor Pathway. Cell Physiol Biochem. 2018;50(4):1332-45.

7. Terms were not used properly in this article. NAF was not common in the description of normal fibroblast. And definitions of positive and high were confused.

We corrected NAF to NF. We also corrected “positive” to” high”. **(on page 2 line 13) (on page 7 line 8-9) (on page 11 line 8)**

Reviewer #3: Dear editors,

In this study, the authors mainly investigated the clinicopathological value of THBS4

In gastric cancer based on the retrospective cohort from their own center. The research methods were statistical analyses of clinical data, including chi-square test, Cox proportional hazards regression models, as well as survival curves. The endpoint parameter was overall survival (OS). The article was well written, and focused on practical clinical aspects. While some problems still existed and should be improved.

1. The exact correlation of α-SMA or CAFs and THBS4 should be explained more apparently both in Introduction and Discussion.

THBS4-high expression in stroma was significantly associated with higher αSMA expression. Forester et al. showed THBS4 is expressed on CAFs which are stimulated by diffuse gastric cancer cells. These findings suggest that CAFs with THBS4 expression might be activated by diffuse gastric cancer cells. Moreover, multivariate analysis for overall survival showed that not αSMA expression on CAFs but THBS4 expression on CAFs was an independent prognostic factor. CAFs with THBS4 expression might be associated with malignant potential of gastric cancer cells. We added these comments in the text. **(Table 1) (Table 2) (on page 3 line 14-16) (on page 4 line 28-29) (on page 7 line 22)** **(on page 10 line 8) (on page 12 line 7-8)** **(on page 12 line 12-20)**

Forster S, Gretschel S, Jons T, Yashiro M, Kemmner W. THBS4, a novel stromal molecule of diffuse-type gastric adenocarcinomas, identified by transcriptome-wide expression profiling. Mod Pathol. 2011;24(10):1390-403.

2. The qualities of all K-M curves were poor. We believe this problem should be focused since key results of this study were reflected herein. Some normative forms could be referenced, for instance, ANNALS OF ONCOLOGY.

In accordance with the Reviewer’s comment, we renew the Kaplan-Meier curve referencing ANNALS OF ONCOLOGY. Graph size and line style were changed and censors were added.

3. Information from Figure 3 was too simple to explain exact relation between the expression of THBS4 and α-SMA or CAFs.

We added the comments of Fig 3 more in detail, as follows. **(on page 11 line 14-18)**

**Figure legends**

**Fig 3. The picture of CAFs and NFs stained with Podoplanin and the western blot analysis of THBS4. (A), Representative picture of Podoplanin staining.** Podoplanin was mainly stained at the cytoplasm fibroblasts. The expression level of Podoplanin was higher in CAFs, in compared with that in NFs. (B). **THBS4 expression.** CAFs expressed THBS4, but NFs and cancer cells did not.

4. References should be updated by using articles in recent 5 years.

We updated references using recent articles.

**Reviewer #4**

Thank you very much for the careful review of the Reviewer #4. We correct several points according to the descriptions by the Reviewer #4, as follows.

I have some comments with respect to the methodology of CAFs and NAFs isolation for western blot analysis. The authors have not elaborated on the specific markers that were used to identify these from the samples.

We used the specific markers, Podoplanin, to identify CAFs and NFs in Figure 3A. We also added the comments in the materials and methods and the results. **(on page 5 line 12-16)**
